# Editorial: Roots of resilience: exploring community-focused mental health through place

**DOI:** 10.3389/fpubh.2026.1996639

**Published:** 2026-09-28

**Authors:** Salim Al-Huseini, Melanie Rees-Roberts, Nagina Khan

**Affiliations:** 1 Department of Rehabilitation, AL Masarra Hospital, Ministry of Health, Muscat, Oman; 2 Centre for Health Services Studies, London and Southeast University Group, Canterbury, United Kingdom; 3 Centre for Health Services Studies, London and Southeast University Group, University of Kent, Canterbury, United Kingdom

**Keywords:** community mental health, mental health, place, prevention, workforce

## 1 Introduction

Mental health represents one of the most significant public health challenges globally. Recent information from the World Health Organization (1) has estimated that more than 1 billion people will live with a mental disorder in 2025, with anxiety and depressive disorders among the most common conditions (2). Poor mental health also has devastating consequences, with suicide affecting people in all countries and contexts and as a particularly concerning cause of death among young people (1).

The economic impact of poor mental health is significant, with mental disorders accounting for the second leading cause of years lived with disability globally (3). Importantly, this burden is not distributed equally. Depressive, anxiety and eating disorders are more prevalent among females and differ with age (2). The burden among adolescents and young adults is particularly concerning in some countries, with recent global estimates that 279 million people aged 10–24 years were living with a mental disorder in 2021, with females aged 15–24 experiencing particularly high levels of disability (2, 4).

Compounding these worrying prevalence rates globally, mental health systems across the world are marked by large gaps and imbalances in resources, services, information and research, as well as governance, especially in low-income countries (1, 3). Alongside the fact that most people living with a mental health condition do not receive formal care (1), there is a need to think differently for better prevention, diagnosis, care and recovery.

### 1.1 Moving beyond the medical model: mental wellbeing, community and place

As Editors of this Research Topic, we believe the increasing prevalence of mental health problems and ongoing inequities in access to care necessitate a broader conceptualization and approach to mental health. While diagnosis and evidence-based treatment are crucial for individuals with mental disorders, they should not be the exclusive framework for population mental health. Mental health is also shaped throughout life by social relationships, living conditions, cultural context, and opportunities for participation and inclusion. This expanded perspective aligns with the World Health Organization's call for mental health systems that integrate treatment with promotion, prevention, action on social and environmental determinants, and community-based care (3, 5).

This vision holds relevance for populations experiencing social and structural vulnerability. Factors such as poverty, unemployment, insecure housing, discrimination, violence, and social isolation can adversely affect mental health while simultaneously creating barriers to appropriate support (6, 7). Addressing these challenges therefore requires coordinated action across health, social care, education, employment, housing, and community sectors. Fundamentally, community should be understood as more than a setting for service delivery. Communities can provide social connection, belonging, practical support, and opportunities for inclusion and meaningful participation, which are important components of recovery and wellbeing (8, 9). A place- and context-sensitive approach can therefore enable mental health services to respond more effectively to local needs, cultural realities, and existing community strengths.

The articles within this Research Topic exemplify this broader vision by showcasing innovative approaches that move beyond conventional clinical treatment. They focus instead on prevention, social support, community participation, and context-responsive care. Collectively, these articles demonstrate a shift from solely asking how mental disorders can be treated more effectively to considering how systems and communities can foster conditions that promote wellbeing, support recovery, reduce inequalities, and enable meaningful societal participation.

## 2 How the articles in this topic demonstrate innovation in this wider vision for mental health care across prevention, social support and context, as well as treatment

### 2.1 Novel approaches to community-based care

The contributions to this Research Topic illustrate how community mental health can move beyond conventional service models by bringing care closer to people's everyday lives and connecting clinical support with social participation and recovery. In Spain, Besoaín-Cornejo et al., Exploring lived experiences in home-based psychiatric care, examined crisis resolution and home treatment from the perspectives of service users, families and professionals, illustrating how intensive multidisciplinary psychiatric care can be delivered within people's homes while maintaining a person-centered approach. Similarly, Adam et al.'s 3for1 intervention program, short-term psychotherapy, job coaching, and peer support, demonstrates an explicitly cross-sectoral model in which psychotherapy, employment support and peer support are integrated for unemployed people experiencing psychological distress. Rather than treating mental health independently of social circumstances, the model addresses psychological wellbeing alongside employment and social participation. The Research Topic also extends community care beyond clinical services through Simpson et al., Going beyond: recovery and homelessness supported housing provision, highlighting supported housing as an environment in which recovery can be experienced through stability, autonomy and everyday community life. Likewise, Boumans et al., Recovery through creativity, demonstrates how arts-based initiatives can provide meaningful spaces for recovery and social connection beyond conventional treatment settings. Finally, Madkour et al., Community-based mental health centers in Ukraine, illustrate the importance of developing and evaluating community mental health centers within real-world contexts. Collectively, these contributions suggest that innovation lies not simply in delivering psychiatric interventions outside hospitals, but in integrating treatment with home, work, housing, creativity and community life.

### 2.2 Wider determinants of health—the roots of resilience

A key message emerging across this Research Topic is that mental health and wellbeing are inseparable from the wider social, economic, physical and environmental conditions and pressures in which people live. A substantial body of evidence demonstrates that factors including personal characteristics alongside experiences (including poverty, unemployment, insecure or inadequate housing, discrimination, social exclusion and adverse environments/experiences) shape and influence both mental health and opportunities for recovery (6, 7). Addressing wider determinants does not mean replacing clinical care; rather, it means recognizing that clinical interventions operate within social and wider contexts that can either enable or constrain their effectiveness. Hamilton et al. highlight this through the experiences of families and carers within community-based psychiatric services, reminding us that the effects of mental ill-health and the resources required for recovery extend beyond the individual service user. The contributing articles in this Research Topic reflect the need for a diverse and comprehensive understanding of support across clinical and wider determinants of health, as demonstrated by Adam et al. through an integrated model combining psychotherapy, employment support and peer support. The impact of wider determinants often extends beyond simple basic needs, as highlighted by Simpson et al. who showed how housing is more than a physical environment for people experiencing homelessness or recovering from substance use, who frequently experience mental health challenges. They describe the importance of safety, autonomy, relationships, activities and community in creating conditions that support recovery.

Wider determinants of health are often linked to “place” across human and natural environments and how we interact with these, with physical health, safety, and social connection being important for mental wellbeing. Chandra Mouli et al. explore neighborhood greenspace using mobility data across nine US metropolitan areas, highlighting the potential importance of how people actually interact with local environments rather than simply whether greenspace is geographically available. Place-based mental health approaches therefore offer an opportunity to connect clinical provision with action on the wider determinants that generate vulnerability, often drawing in existing community assets and sources of resilience to support wellbeing and recovery. Mogaji et al. illustrated how community-based participatory approaches can bring local knowledge and priorities into the identification and development of interventions, while Boumans et al., show how participation in asset-based arts recovery initiatives can create connection, meaning and social participation to support wellbeing. These findings also reinforce the importance of prevention as part of a community-focused approach to mental health. Promoting social connection, participation, resilience and supportive environments may help build protective factors before mental health difficulties become more severe, alongside ensuring timely access to effective treatment when it is needed.

## 3 Future needs to support change

### 3.1 Capacity for change in the system

Sustainable transformation depends not only on expanding community services but also on strengthening mental health systems to effectively integrate, evaluate, and scale community-based approaches. This requires multidisciplinary teams, strong leadership, comprehensive workforce training and supervision, sustainable financing, and clear pathways connecting specialist, primary, social, and community care (6). The changing nature of community mental health also has implications for the workforce. Effective community-focused care requires professionals to work alongside people with lived experience, peer workers, families, community organizations and services beyond the health sector. This means developing skills in collaboration, co-production and community engagement alongside conventional clinical competencies. The examples within this Research Topic, including the 3for1 model described by Adam et al. and the Veteran Voices and Visions groups, demonstrate the value of bringing different forms of expertise and support together. Workforce development therefore needs to create the skills, roles and organizational conditions that allow these partnerships to develop.

These systems must be adaptable, able to incorporate community insights and respond dynamically to local needs. The meaningful involvement of individuals with lived experience and community stakeholders in planning, implementing, and evaluating services is crucial for enhancing the relevance and responsiveness of care. Innovation also demands appropriate governance frameworks, rigorous outcome measurement, and established mechanisms for identifying and scaling effective approaches, all while maintaining local contextual relevance (7). Importantly, system performance assessment should go beyond metrics like hospital utilization and symptom reduction. It should include broader indicators such as recovery, functional capacity, quality of life, autonomy, and social participation. Such outcomes provide a more holistic appraisal of whether mental health services genuinely empower individuals to lead meaningful and socially integrated lives (10).

The inherent challenge lies in building systems that operate collaboratively with communities and are embedded within specific localities. This establishes community-based, person-centered care as a foundational organizing principle rather than merely a supplementary service layer.

### 3.2 Equity considerations within change

If mental health systems are to become community-focused, equity must be central to how services and interventions are modified, designed, delivered and evaluated to reduce health inequalities that exist. The unequal distribution of mental health problems is shaped by individual circumstances, inequalities in access to appropriate, acceptable and timely support as well as the wider conditions in which people live (6, 7). A community-based service is not necessarily an equitable service; therefore, system change will need to be aware and proactive to combat inequality. Bringing care closer to communities will have limited impact if the people most affected by disadvantage remain unable to access, trust or benefit from it. An equity-focused approach should move beyond measuring whether services are available to asking who benefits, who does not, and why.

## 4 Conclusion

The articles in this Research Topic collectively demonstrate that innovation in mental healthcare extends beyond new treatments to encompass how care is organized, delivered, and connected to people's social and community contexts. We believe sustainable progress necessitates systems that integrate prevention, treatment, social support, recovery, and community participation, whilst also responding to local needs. Place, social determinants, lived experience, and equity also need to shape what is provided, for whom, and how success is judged. The challenge ahead lies in translating promising approaches into sustainable practice by strengthening system capacity, fostering meaningful community engagement, and enhancing cross-sector collaboration. Centring mental health systems around people, communities, and context offers a more inclusive and sustainable pathway toward mental health and wellbeing.
